# Controllable Moderate Heating Enhances the Therapeutic Efficacy of Irreversible Electroporation for Pancreatic Cancer

**DOI:** 10.1038/s41598-017-12227-4

**Published:** 2017-09-18

**Authors:** Chelsea M. Edelblute, James Hornef, Niculina I. Burcus, Thomas Norman, Stephen J. Beebe, Karl Schoenbach, Richard Heller, Chunqi Jiang, Siqi Guo

**Affiliations:** 1 Frank Reidy Research Center for Bioelectrics, Old Dominion University, Norfolk, Virginia 23508 USA; 2 Department of Electrical & Computer Engineering, Batten College of Engineering & Technology, Old Dominion University, Norfolk, Virginia 23508 USA

## Abstract

Irreversible electroporation (IRE) as a non-thermal tumor ablation technology has been studied for the treatment of pancreatic carcinoma and has shown a significant survival benefit. We discovered that moderate heating (MH) at 43 °C for 1-2 minutes significantly enhanced *ex vivo* IRE tumor ablation of Pan02 cells by 5.67-fold at 750 V/cm and by 1.67-fold at 1500 V/cm. This amount of heating alone did not cause cell death. An integrated IRE system with controllable laser heating and tumor impedance monitoring was developed to treat mouse ectopic pancreatic cancer. With this novel IRE system, we were able to heat and maintain the temperature of a targeted tumor area at 42 °C during IRE treatment. Pre-heating the tumor greatly reduced the impedance of tumor and its fluctuation. Most importantly, MHIRE has been demonstrated to significantly extend median survival and achieve a high rate of complete tumor regression. Median survival was 43, 46 and 84 days, for control, IRE with 100 μs, 1 Hz, 90 pulses and electric fields 2000–2500 V/cm and MHIRE treatment respectively. 55.6% of tumor-bearing mice treated with MHIRE were tumor-free, whereas complete tumor regression was not observed in the control and IRE treatment groups.

## Introduction

Pancreatic cancer, with an overall 5-year survival of 5%^1^, is one of most deadly cancer types. The mortality rate from pancreatic cancer has increased in both Europe^2^ and the US^3^. While research and clinical trials have enhanced knowledge about this disease, there is limited progress on improving patient survival. Late stage disease with micro- or macro-metastases, large tumor burden with multiple signaling pathways involved, and molecular heterogeneity^4^ make conventional therapies rarely successful and current targeting therapies, which often target a single pathway, insufficient. Pancreatic cancer is notoriously resistant to chemotherapy as are cancer stem cells, which are common in pancreatic cancer^5,6^. New effective therapeutic strategies are desperately needed for this deadly disease.

In the last decade Irreversible electroporation (IRE) as a non-thermal ablation technology has been developed and studied in animal models for normal/diseased tissue^7–9^ and tumor ablation^10,11^. Recently IRE has reached clinical trials (ClinicalTrials.gov) for liver^12^, rectal, renal^13^, stomach, pancreatic cancers^14–17^, etc. most of which are unresectable or metastatic cancers. Survival extension of 6–8 months was achieved in patients treated with IRE. This a significant improvement for locally advanced pancreatic cancer^18^. A systematic review showed significant survival benefits when treating advanced pancreatic cancer with IRE^19^. IRE is seen as a safer ablation technology than the alternative: hyperthermia, which is associated with high morbidity and mortality due to thermal damage to adjacent structures. However, incomplete ablation with local recurrence^18,20–22^ as well as major complications^17,18,21^ associated with IRE protocols and needle placement are two major issues that limit the potential of IRE. Obviously, technology or approaches, which can improve complete tumor ablation and/or reduce adverse events, will benefit patients and potentially broaden the applications of IRE.

Temperature dependent electric properties of biological tissues have been studied for over three decades^23^. Our hypothesis is that a moderate increase in the temperature of the target tumor can decrease tumor impedance, thereby sensitizing the target tumor for IRE tumor ablation. To test this hypothesis, a 3D agarose gel cell-culture model was studied for *ex vivo* IRE tumor ablation. A novel electric pulse delivery system which includes an infrared laser for controlled heating of the treatment area has been developed. The system contains also components which allow monitoring temperature and impedance changes of the treated tissue. Therapeutic efficacy of moderate heating IRE (MHIRE) was evaluated in an ectopic murine pancreatic adenocarcinoma model.

## Results

### Development of an integrated controllable laser heating IRE system

**Figure 1:**
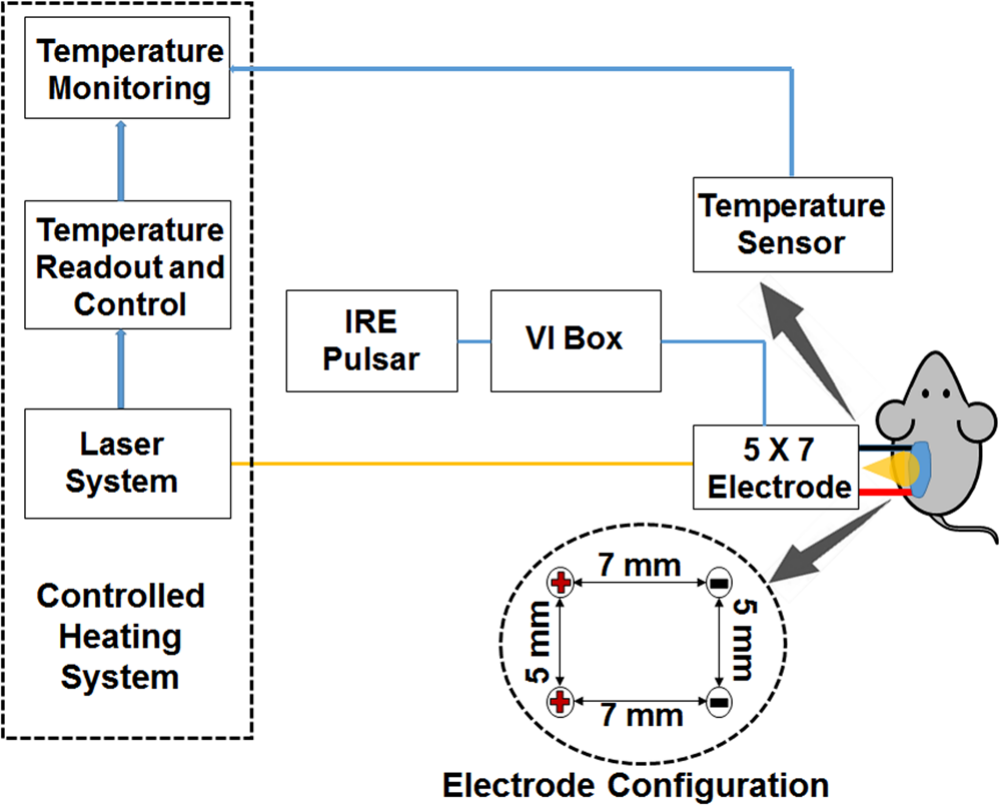
Schematic diagram of MHIRE system. Laser control system consists of a laser power supply, a laser optic fiber placed at the center of a four-needle electrode, a thermopile or thermocouple with feedback of tumor temperature and a laptop with temperature monitoring and laser control software. Electric pulses are generated by a BTX ECM 830 Square Wave Electroporation System (IRE Pulsar) and delivered with a four-needle electrode. The configuration of electrode is shown. Impedance analysis is done with a voltage probe, current-viewing resistor, which we call a VI box, and a multi-channel oscilloscope.

We have developed a novel electric pulse (EP) delivery system that can heat and maintain certain surface temperatures on target tissue/tumor, deliver EPs and monitor the impedance in real time. This system consists of three units as seen in Fig. 1, an electric pulse supplier, a laser heating and control system and a voltage/current monitoring system. In this study, we used an ECM 830 Square Wave Electroporation System as EP supplier. The laser heating and control system was made up of a laser power supply, a laser optic fiber, a thermopile temperature sensor and a custom made laser control box connected to a laptop computer, allowing the user to set a target surface temperature and monitor the surface temperature in real time. Both the laser optic fiber and a thermopile sensor are integrated into a four-needle electrode, and both were able to operate while pulsing. The temperature control system was isolated electrically, in order to prevent electrical interference from the high voltage pulsing, which allowed it to be used during the pulsing treatment. Before testing, a thermocouple was inserted into tissue or tumor in order to correlate the surface temperature with the internal tissue temperature for calibration of laser control model. The temporal development of voltage across the treated tissue between the electrodes and the current are monitored with a multi-channel oscilloscope and the temporal change in impedance was deduced from the voltage and current data.

### Enlargement of *ex vivo* tumor ablation of IRE with a controllably moderate heating

**Figure 2:**
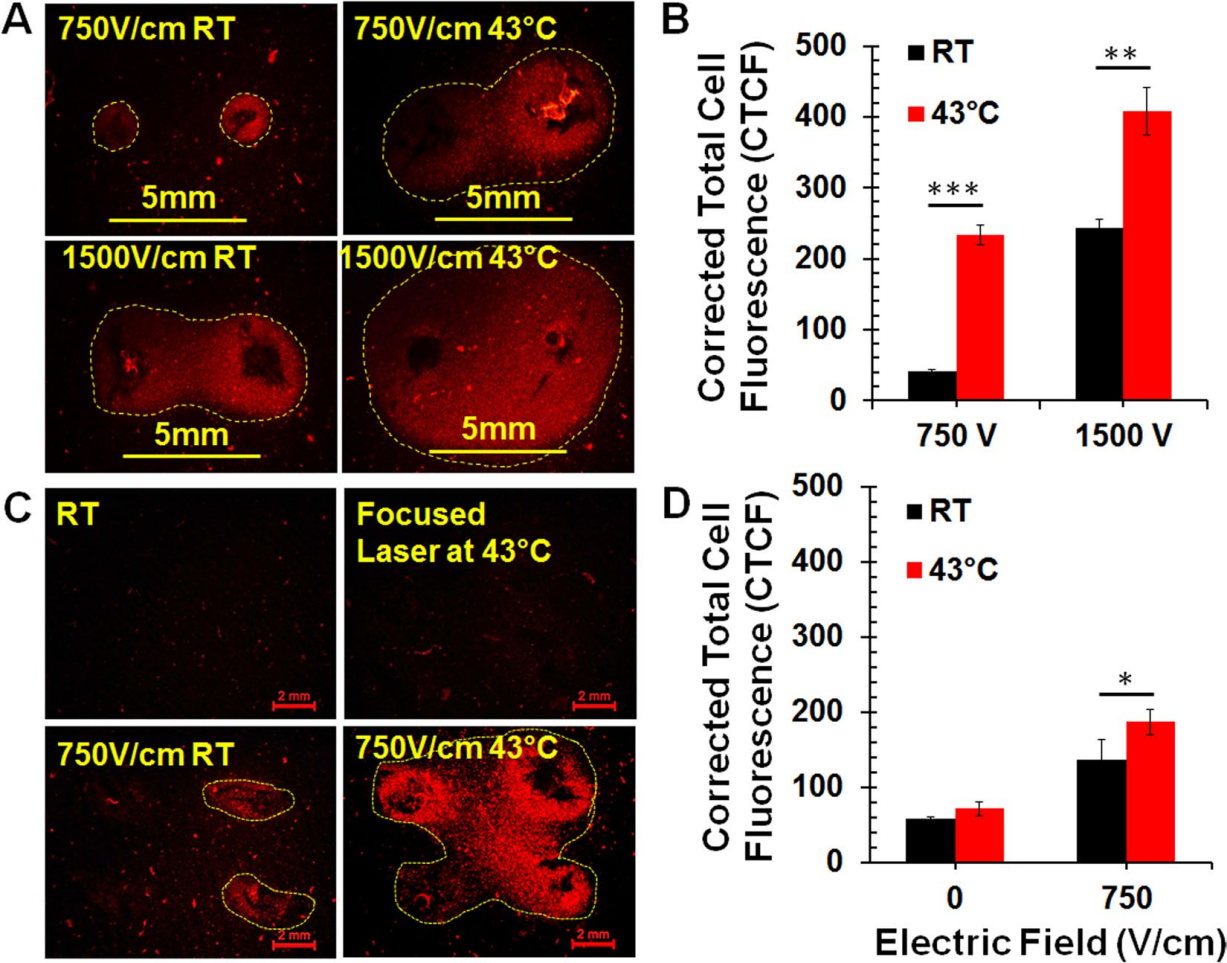
Enlargement of *ex vivo* tumor ablation zone with moderate heating IRE. A 3D agarose gel Pan02 tumor model was treated by IRE with a two-needle (**A**,**B**) or a four-needle (**C**,**D**) electrode. Area with red color was zone of dead cells indicated by Propidium Idide (PI) staining. IRE parameters: pulse duration 100 µs, frequency 1 Hz, pulse number 80 and applied electric fields 750 V or 1500 V/cm. RT: room temperature; 43 °C: samples preheated at 43 °C by heat block (**A**,**B**) or laser (**C**,**D**). 5 mm or 2 mm: Scale bar. Corrected Total Cell Fluorescence (CTCF), as a measure of cell death, was analyzed by ImageJ software. CTCF = integrated density – (area x mean fluorescence of background). Number of repetition (n) per treatment group = 3–4. *p < 0.05, **p < 0.01 and ***p < 0.001 for RT vs 43 °C by t-test.

First, we compared IRE tumor ablation at room temperature or pre-heating samples on a heat block. A 3D agarose cell-culture model was utilized to study *ex vivo* IRE tumor ablation. Pre-heating Pan02 tumor cells to 43 °C can significantly enhance IRE tumor ablation with a two-needle electrode, while tumor cells with 2 minutes of heating at 43 °C did not result in any observable cell death. The ablation zone, indicated by integrated fluorescence density, increased 5.67-fold with IRE pulses at an electric field of 750 V/cm (p < 0.001) and 1.67-fold with IRE pulses at an electric field of 1500 V/cm (p < 0.01) when cells were preheated to 43 °C. (Fig. 2A and B).

The same phenomenon occurs when focused heating with a laser was applied to the treated area. As seen in Fig. 2C and D, pre-heating the treatment area to 43 °C using our novel four-needle electrode, significantly enlarged the tumor ablation zone. In contrast to IRE with 750 V/cm alone, moderate heating IRE increased the integrated fluorescence density 1.4-fold (p < 0.05).

### Simulation of electric field distribution and laser beam profile

**Figure 3:**
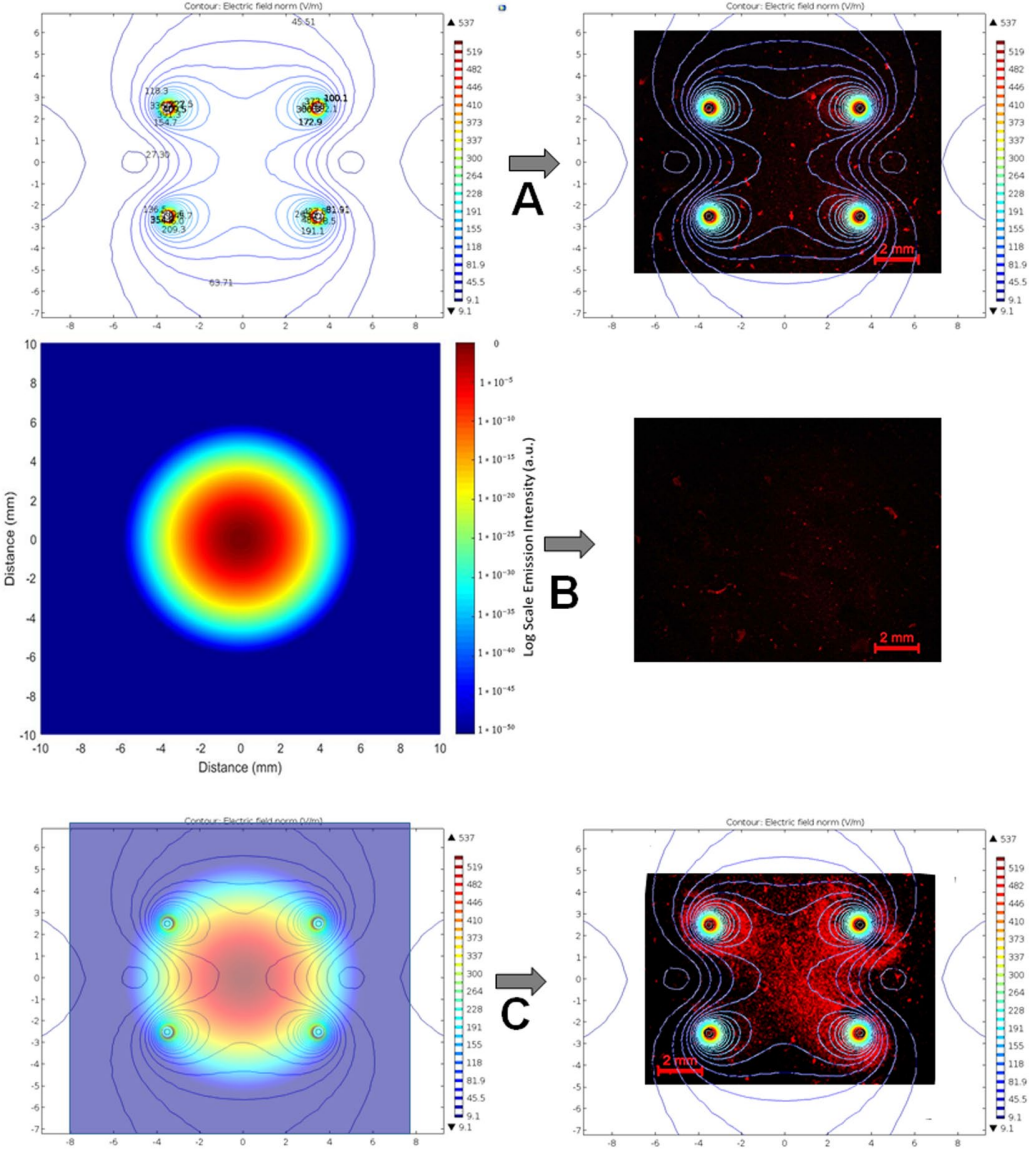
A complementation between electric field distribution and laser beam profile for MHIRE tumor treatment. (**A**) Electric field distribution (left) of a 5 × 7 mm four-needle electrode with 525 V applied between the anode and cathode (corresponding to 750 V/cm), and the electric field-only *in vitro* result (right); (**B**) Normalized laser beam profile in log scale (left) and the corresponding heating-only result (right); (**C**) Combination of the electric field and the laser beam profile (left) and the significantly improved, more uniformly treated outcome. Both the electric field distribution and laser beam Gaussian profiling were from computational results using Comsol Multiphyics and Matlab softwares. The electric field is strongest around the needles, which surround the tumor. The intensity of the laser is highest at the center, resulting in a higher temperature in the center of the tumor. A 3D agarose gel Pan02 tumor model was used for the *in vitro* study. Area with red color was zone of dead cells indicated by Propidium Iodide (PI) staining.

Electric field distribution of the 5 × 7 mm four-needle electrode with 525 V applied between the left and right electrodes, corresponding to an average field strength of 750 V/cm (for a parallel-plate electrode), was calculated, as shown in Fig. 3A (left). The electric field strength, expressed in color contour and labeled in kV/cm, decreased rapidly with distance away from each electrode surface; within 0.5 mm radial distance from the electrode, the electric field decreases from >2 kV/cm to 600–700 V/cm. Overlapping of the electric field distribution with the *in vitro* cell fluorescence image shows that the region of cell death corresponds to an electric field > 645 V/cm (Fig. 3A, right). The use of the four-needle array is able to increase the region of treatment by four times, compared to a single needle electrode arrangement. However, the center and the middle regions between the needles have relatively weaker field (~500 V/cm in this case). Application of moderate heating is able to compensate for the lower field strength, and enhance the ablation zone as shown in Fig. 3C (right).

Calibration of the laser irradiation-based heating system was first performed on pig skin tissue samples under similar conditions as the tumor ablation treatment, and the optimal settings of the heating system were identified to obtain 43 °C on the target surface. As the temperature sensor, i.e. the thermopile, only provides a mean temperature based on the viewing region, the temperature distribution as a result of instant heating distribution is unknown from these measurements.

In order to provide a more accurate view on how the tumor has been heated during the treatment, the profile of the laser beam was measured using the knife-edge technique^24^. A sharp razor blade was moved transversely across the beam in an increment of 5 µm and the transmitted power after the knife-edge was recorded by a power meter. The trend line collected using this technique results in the 2D Gaussian profile (Fig. 3B) by integrating of the displacement of the knife-edge. In order to get the width of the beam, the derivative of the trend data was taken and a 2^nd^ order Gaussian fit was applied to the resulting data. In this way, the laser beam profile was identified to have a beam width of about 2 mm (from 10–90% value). We were able to create a 3D intensity plot following the Gaussian distribution using the beam width (Fig. S1). The irradiation of the laser beam was normalized after applying a logarithmic scale, as indicated in the color mapping of Fig. 3B.

### Temperature and impedance monitoring of tumor with the novel controllably heating IRE system

**Figure 4:**
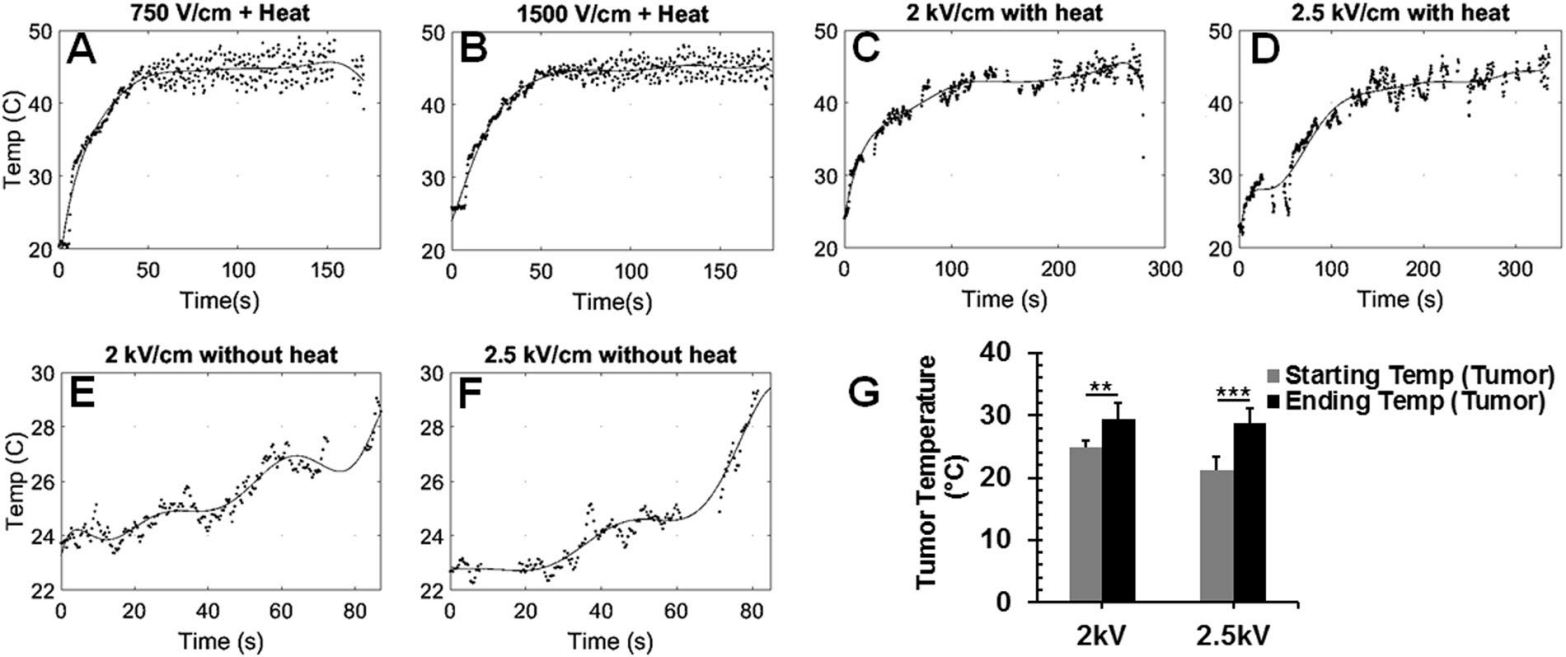
Temperature monitoring during *in vivo* MHIRE treatment. The temperature trends were taken by the thermopile system using the four needle electrode (5 × 7 mm) during treatment. Target surface temperature was set to 45 °C, which allows the surface tumor temperature to reach and be maintained at 43 °C on average. Heating time was set to 60 seconds and then the electric field was applied while maintaining the heating. IRE parameters: 100 µs pulse width, 90 pulses, frequency of 1 Hz and applied electric fields 750 V/cm (**A**), 1.5 kV/cm (**B**), 2 kV/cm (**C**,**E**,**G**) and 2.5 kV/cm (**D**,**F**,**G**). The slower temperature increase and increased variations shown in (**C**,**D**) were due to ultrasonic gel to reduce electrical breakdown and the electrical breakdown that did occur respectively. Using this system, a heating effect was observed with pulsing alone (**E**–**G**). One representative sample from 4 to 8 tumors is shown here.

With our MHIRE system, the surface temperature of the target tumor can be monitored by a thermopile temperature sensor. In order to make sure there is a temperature increase occurring inside the tumor and not just on the surface, a pretest was done to determine the laser control setting necessary to heat the inside of the tumor to the target temperature. A thin thermocouple with tip size 2.5 × 2.5 ± 0.1 mm was inserted in the bottom part of tumor and the time it took the reading to reach 42 °C was recorded. It took 20–60 seconds for the internal tumor temperature to reach 42 °C when the surface target temperature was set to 45 °C by laser heating. So, based on the calibration results, we decided that pre-heating tumor for 60 seconds before EPs were delivered would allow the inside of the tumor to be at the correct internal temperature. Each representative surface temperature curve for four MHIRE protocols are shown in Fig. 4. An average surface temperature of 44–45 °C was achieved with a fluctuation within 42 °C to 47 °C. The rise time to reach 45 °C was varied for each tumor because of the difference in tumor shape and whether there was a center necrosis or ulceration. In general, an ulceration will slow down the heating process. Noticeably, a much longer pre-heating time was needed for MHIRE with 2 kV/cm or 2.5 kV/cm (Fig. 4C,D). This is because ultrasound gel was applied on the tumor surface to prevent potential electrical breakdown. The ultrasound gel caused the surface temperature to decrease and the heating time to increase, since the laser had to heat the gel before heating the tumor itself.

With this novel design, a temperature increase of the tumor due to IRE alone was observed. Maximal temperature increase of the tumor was 7.5 °C for IRE with 2.5 kV/cm (p =  < 0.001) and 4.7 °C for IRE with 2 kV/cm (p = 0.002) (Fig. 4E–G). Because of the relative low base temperature of the tumor, which was between 23 °C to 27 °C, especially when the dielectric gel was applied, the temperature increase due to IRE is likely not to impact the tumor ablation.

**Figure 5:**
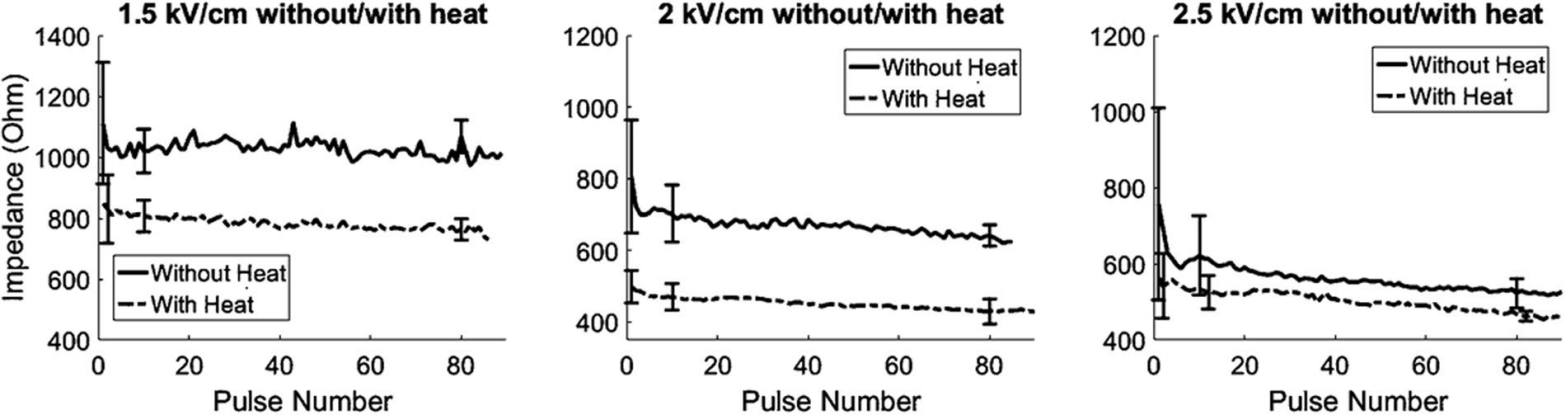
Tumor impedance measurement during MHIRE treatment. Impedance trends were taken from the voltage probe and current-viewing resistor readings over the 90 pulses applied. Each data point represents an average impedance reading of 4 to 5 tumors for that pulse. IRE parameters: 100 µs pulse width, 90 pulses, frequency of 1 Hz and applied electric fields of 1.5 kV/cm (left), 2 kV/cm (middle) and 2.5 kV/cm (right). Standard deviations for the 1^st^, 10^th^ and 80^th^ pulses are indicated with error bars.

Impedance monitoring during IRE or MHIRE treatment was done with a combination of a voltage probe and current-viewing resistor, which used the voltage and current at the load, or tumor/tissue, to determine the resistance at that pulse. From the impedance curves (Fig. 5), we know that the baseline impedance of each tumor varied but a moderate heat could reduce it by 200–300 Ω or 15–38% of the baseline impedance, 750 to 1100 Ω. The reduction of the impedance was correlated to strength of the electric fields. The higher electric field, the more the impedance was reduced by the end of the treatment. Additionally, it appears that MH also reduces the fluctuation of impedance changes, which may indicate that MH enhances uniformity of the tumor dielectric property. The average drop of impedance was 39.1 to 46.6% for MHIRE with 2000 to 2500 V/cm and 22.4 to 30.5% for IRE with the same electric field. Because complete tumor ablation only occurred with MHIRE group, this indicates that a large reduction of impedance maybe necessary for complete tumor ablation.

### Enhancement of the therapeutic efficacy of IRE with MH for ectopic pancreatic cancer

**Figure 6:**
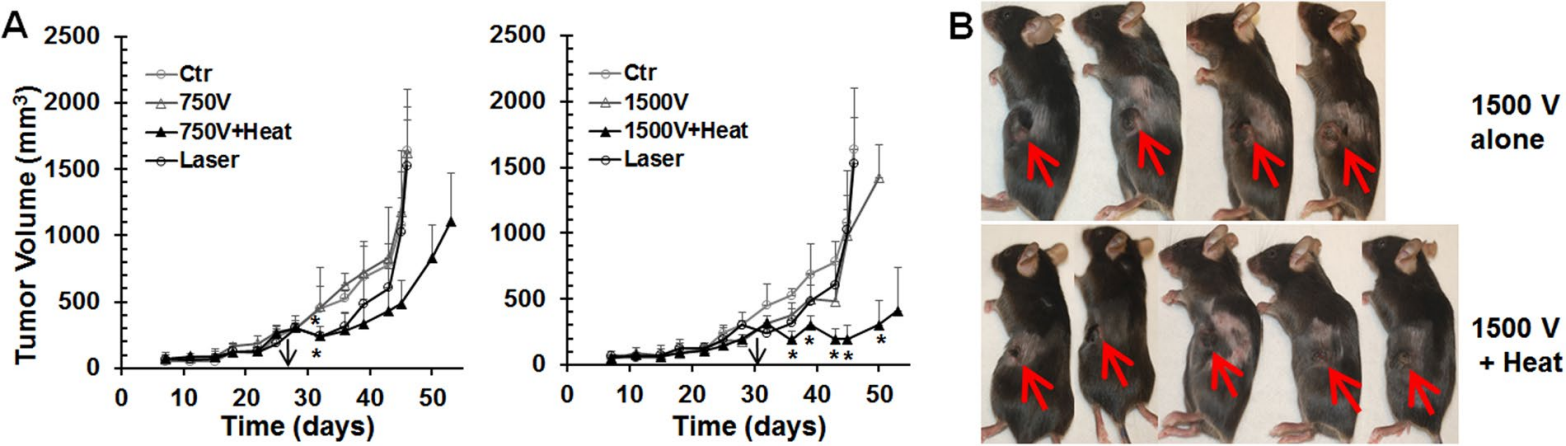
Pancreatic tumor growth after IRE or MHIRE treatment. Pan02 pancreatic tumors with the size of 8 to 10 mm were treated with IRE or MHIRE at day 28 or 31 indicated by black arrow. IRE parameters: pulse duration 100 µs, frequency 1 Hz, pulse number 90 and applied electric fields 750 V or 1500 V/cm. Ctr: no treatment (n = 4 mice per treatment group); Laser: tumor heated with laser at 42 °C for 2 minutes; 750 V, 1500 V: IRE at 750 V/cm or 1500 V/cm (n = 4 mice per treatment group); 750 V + Heat or 1500 V + Heat: tumor preheated with laser at 42 °C with IRE at 750 V/cm (n = 7 mice per treatment group) or 1500 V/cm (n = 8 mice per treatment group). A: tumor growth curve. B: Pictures taken at post-treatment day 2. *p < 0.05, or p < 0.01 or p < 0.001 for MHIRE vs IRE or Ctr by One Way ANOVA.

First, we treated the tumors with either IRE or MHIRE with parameters: 100µs pulse width, 1 pulse per second, 90 pulses and 750 V/cm or 1500 V/cm. Both IRE treatments alone had little impact on the tumor growth. However, a synergistic effect was seen in the IRE treatment when the tumor was preheated to 42 °C (internal temperature) for both 750 V/cm and 1500 V/cm IRE protocols (Fig. 6A). A significant decrease of tumor size was seen in animals treated with MHIRE at 750 V/cm only at one-time point at post-treatment day 8 (p < 0.05) whereas tumors treated with MHIRE at 1500 V/cm were all significantly smaller than control group or the IRE alone group at all time points on post-treatment days 4, 7, 11, 13 and 14 (p < 0.05, 0.05, 0.05, 0.01 or 0.001, separately). As shown in Fig. 6B, at post-treatment day 2, typical central necrosis of tumor occurred after IRE with 1500 V/cm, whereas a larger tumor necrosis was observed in MHIRE with 1500 V/cm. Nevertheless, no complete tumor regression was obtained under these two IRE or MHIRE protocols.

**Figure 7:**
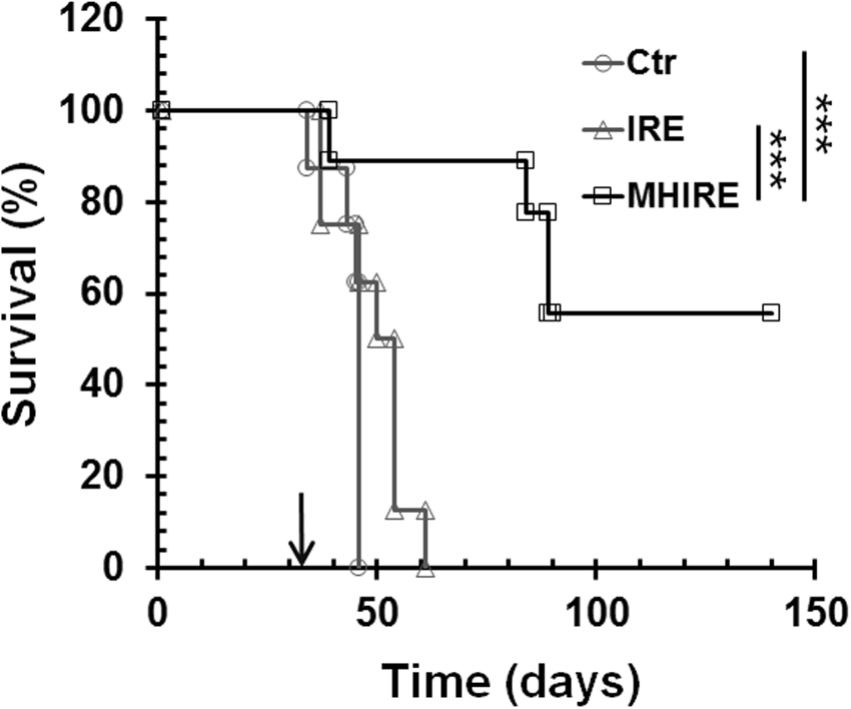
Kaplan-Meier survival curves of mice treated with IRE or MHIRE. Pan02 pancreatic tumors with the size of 8 to 10 mm were treated with IRE or MHIRE at day 31 indicated by arrow. IRE parameters: pulse duration 100 µs, frequency 1 Hz, pulse number 90 and applied electric fields 2000–2500 V/cm. Ctr: no treatment (n = 8 mice per treatment group); IRE: treated with IRE (n = 8 mice per treatment group); MHIRE: tumor preheated with laser at 42 °C with IRE (n = 9 mice per treatment group). ***p < 0.001 for MHIRE vs IRE or Ctr by LogRank test.

We then treated tumors with IRE or MHIRE at elevated electric fields within 2000–2500 V/cm. The Kaplan-Meier survival curves are shown in Fig. 7. Despite the higher electric fields, IRE treatment alone could not achieve tumor free animals. It only extended medial survival for 3 days with 46 days median survival compared to 43 days median survival for the control tumor animals. However, MHIRE significantly extended median survival by almost two times with 84 days compared to control mice (p < 0.001). More importantly, 55.6% of the tumor-bearing mice that were treated with MHIRE were tumor-free.

No severely sick animals were found for IRE or MHIRE treatment at 750–1500 V/cm, however, at high electric field 2000–2500 V/cm 5/13 mice in IRE group and 2/11 mice in MHIRE group were euthanized due to severe dehydration, high pain scores or severe sickness. The causes of serious adverse events were likely related to large tumor necrosis or intestine/adjacent tissue necrosis.

## Discussion

Moderate heating itself is not the same as hyperthermia tumor ablation utilized in other studies. Typical duration of MH at a target temperature of 42–43 °C is 2 minutes (Fig. 4). 43 °C was chosen as a target temperature because elevating the temperature above 43 °C could induce cell death^25^, which we wanted to avoid. Heating tumor cells in a 3D culture model for 2 minutes did not cause any cell death (Fig. 2), and moderate heating alone had a slight impact on *in vivo* tumor growth but no effect on survival and tumor regression (Fig. 6). Compared to 1 hour or longer of hyperthermia treatment^26^, direct effect of our MH on tumor cell death is expected to be minimal or negligible. However, synergistic effect between MH and IRE has been demonstrated in both *ex vivo* tumor ablation and *in vivo* tumor reduction. MH is able to significantly enlarge tumor ablation zone *in vitro* (Fig. 2) and reduce tumor growth *in vivo* (Fig. 6).

A simple explanation for this synergistic effect between MH and IRE is the increase of tumor conductivity, indicated by a large decrease in impedance (Fig. 5). Correspondingly, current is elevated so actual dose of precipitate energy to the tumor is higher for MHIRE than that of IRE alone at the same electric field (Fig. S1). A correlation of impedance decrease and the efficiency of tumor ablation was suggested from clinical data^27^. If this is the case, the efficacy of MHIRE should be equivalent to IRE with an elevated electric field. Giving a 40% increase of tumor conductivity, theoretically MHIRE at 2500 V/cm should be identical to IRE at 3500 V/cm. That means using MHIRE could reduce the electric field necessary to achieve the same level of therapeutic efficacy as IRE. With MHIRE, the focused laser beam heats only tumor tissue. This coupled with a reduction in the applied electric field minimizes damage to surrounding tissues and organs, a disadvantage of current IRE ablation therapies. Another effect of MH is the decrease of impedance fluctuation of the tumor. This phenomena might contribute to more complete tumor ablation. Because a tumor is not a homogenous structure with multiple types of cells and extracellular matrix^28,29^, a heterogeneous impedance map of tumor tissue^30^ is expected and as a result the tumor impedance during IRE treatment fluctuates. Any approach to enhance the uniformity of tumor physical property could result in more tumor cells being exposed to the lethal dose of electric field and consequently a higher rate of complete tumor ablation.

In contrast to other studies of IRE for xenograft murine pancreatic cancer models, identical or similar parameters were adopted but higher complete tumor ablation was achieved in those reports. An extension of medial survival from 42 days in control mice to 88 days with 25% of complete tumor ablation after IRE treatment was reported by Jose *et al*.^31^. In that study, a matched IRE protocol (100 µs, 1 Hz, 2500 V/cm and pulse number 90) was applied but smaller (2–5 mm) xenograft human BxPC-3-luc orthotopic pancreatic tumors in athymic nude mice were treated. Philips *et al*. claimed that 74.4% of complete tumor ablation was obtained histologically, but no survival study was conducted. A large (1–1.5 cm) ectopic human Panc-1 pancreatic cancer in the skin of hind thigh of Balb/c nude mice was treated by a two-needle electrode with a 4 mm gap. IRE parameters were 100 µs, 1800–2000 V and 90 pulses. Though tumor size, choice of electrodes and IRE parameters all influence therapeutic efficacy, the sensitivity of tumor cells to electric pulses and the received dose of electric pulses would determine the fate of tumor cells. Therefore physical properties of the target tumor, especially impedance is a critical factor for IRE treatment. Unfortunately, there was no impedance data from these two reports. In contrast to 750 to 800 Ω impedance (Fig. 5C,D) of PAN02 murine pancreatic cancer in this study, 100 to 120 Ω of human pancreatic cancer was reported by Dr. Martin’s group^27^. The received current or energy dose of human pancreatic cancers is 6-7-fold higher than murine pancreatic cancers. That explains why mouse Pan02 cancer was more resistant with a similar IRE protocol.

It is suggested that IRE is a novel ablation modality better than hyperthermia based ablation technologies to avoid unselective tissue damage and “thermal sink”^32^. Nevertheless, adverse events including severe complications did occur with IRE treatment in clinical trials^14–18,33^. High-grade complications, such as bile leak^15,18^, portal vein thrombosis^15^, duodenal leak^15,18^, pancreatic leak^18^, pancreaticoduodenal fistula^14^, are associated with IRE protocols. Though the imprecise needle placement is thought to be a major cause of those serious complications, the contribution of electric pulses could not be ruled out. A recent study demonstrated that placement of electrodes could cause damage to the adjacent normal tissue without direct needle insertion^34^. Adjacent muscle/skin damage and intestine necrosis (data not present here) was evidenced from necropsy of ectopic pancreatic cancer mice treated with IRE at high electric fields (2000–2500 V/cm) in this study as well. Additionally, 26.2% (17/65)^27^ or 27.8% (15/54)^18^ of local failure or recurrence also limits the benefit of this treatment. Certainly, there is still a space for the improvement of this novel ablation technology. Here we intended to improve the efficiency of the current IRE approach for tumor ablation. An integrated EP delivery system with capacity of controllable, focused tissue heating and multi-parameter monitoring has been constructed and tested in a syngeneic mouse pancreatic cancer model. We have demonstrated that moderate heating significantly enhanced the therapeutic efficacy of IRE for pancreatic cancer ablation. Preliminary data also suggest that MHIRE has the potential to eliminate local tumors at lower electric fields with less serious adverse events. IRE as a novel ablation technology has been studied in clinical trials for liver^35,36^, pancreatic^15,33^, kidney^13,37^ and prostate cancers^38,39^, however, there is no standardized protocols or dose definition and monitoring for completion of treatment. A concern of inadequate delivery of energy that could result in incomplete ablation and consequently increase of residual tumor growth rates^27^ was brought up by Dr. Martin’s group^40^. Standardized delivery of energy was suggested as a solution to improve the safety and efficacy of IRE treatment^40^. Change of tissue resistance was measured and defined as the endpoint of procedures in several groups^17,27,33^. The system built here can be utilized to monitor energy delivery (Fig. S2) and the changes in tumor resistance/impedance. More importantly, it can sensitize the IRE treatment by pre-heating tumor. This feature is particularly important for those tumor tissues with high impedance due to the heterogeneous nature of cancer or the difference of tissue types. In those cases, standardized energy would be hard to achieve with the current IRE protocol. Another advantage of our MHIRE protocol, is the reduction in the applied electric field of IRE.

We have to emphasize that our current MHIRE system is a prototype for performing a proof of concept study. One issue that is commonly raised is how to get uniform temperature increase in the target tumor with laser heating. Thermo-distribution of tumor with laser heating has been studied by other groups^41,42^. A typical bell-shape of thermo-distribution is obtained in tumor or tissue with laser heating^41^. It’s obviously central-focused heating. However, considering that electric field of IRE is never evenly distributed with weak spots at the center among 2 or 4 needle prints (Fig. 3), focused laser heating at the tumor center actually corresponds to the electric field distribution of IRE (Fig. 2C). Significant cell death at the center spot with *ex vivo* tumor ablation of MHIRE (Fig. 2C) may ease the concern of the non-uniformity of laser heating. Currently, we are able to treat a target tumor with a size of 8–10 mm, which is not big enough for most clinical tumors. Technically, we can modify the electrode with additional optic lens, increase of needle gap and length to treat 2-3 cm tumor. However, laser heating might be an issue for deep, large tumor tissue. In this case, other heat sources, such as focused ultrasound^43^, microwave^44^ or radiofrequency^45^, could be utilized to pre-heat and maintain moderate temperature increases of large tumor tissue. Another issue is to heat and maintain tumors encasing large vessels. In this case, more complicated designs or even systemic hyperthermia could be adopted to elevate the temperature of blood or the whole body.

In summary, we have developed an integrated MHIRE system with controllable tumor heating, multi-parameter monitoring and electric pulse delivery. We found that the impedance of preheated tumor was greatly reduced as well as its fluctuation. The ablation zone of tumor was significantly enlarged after preheated to 43 °C which alone could not cause any cell death. IRE alone was not sufficient to ablate Pan02 pancreatic cancer at both low and high electric fields, whereas MHIRE significantly reduced tumor size at a lower electric field and resulted in high rate of complete tumor regression at a higher electric field. MHIRE may have the potential to enhance IRE tumor ablation at lower voltages.

This is a first study that has demonstrated a synergistic effect between moderate heating and IRE for tumor ablation and animal survival. This novel concept and technology has the potential to improve therapeutic efficacy of IRE for local tumor eradication.

## Materials and Methods

### Materials and Instruments

Class 4 Laser Power Supply, Wavelength: 980 nm, Output Power: >8 W (Lasermate Group Inc.), a laser optic fiber Model: M79L005 (Thor Labs Inc.), a thermopile based temperature sensor Model: ZTP-135SR model (Amphenol Advanced Sensors), Type K Thermocouple (REOTEMP Instrument Corporation), oscilloscope Model Waverunner 64xi (Teledyne LeCroy), laptop computer Lenovo ThinkPad: I7 Core laptop computer (Lenovo) and ECM 830 Square Wave Electroporation System (BTX-Harvard Apparatus) was purchased from the corresponding vendor.

### Reagents

Low-gelling temperature agarose (Sigma-Aldrich), Propidium iodide solution (Sigma-Aldrich), Bulter Schien animal health aquasonic ultrasound gel (Fisher Scientific), Carprofen (Rimadyl® Injectable, 50 mg/ml) (Patterson Companies) was purchased separately from the corresponding vendor.

### Cell lines

A murine pancreatic adenocarcinoma Pan02 cell line was obtained from the Division of Cancer Treatment and Diagnosis (DCTD), NCI and maintained in RPMI-1640 (ATCC^®^ 30–2001^TM^) supplemented with 10% FBS (Atlantic Biological), 100 IU of penicillin and 100 µg/ml streptomycin. Cells were kept in a 37 °C incubator supplied with 5% CO_2_.

### Mice and tumor models

Female C57BL/6 mice (6–8 weeks of age) were purchased from Jackson Laboratory or Harlan Laboratories. Mice were injected with 1 × 10^6^ Pan02 cells in 50 μL Dulbecco’s phosphate buffered saline (DPBS) on the left flank. The size of primary tumor was assessed by digital calipers twice a week. Tumor volume was determined using the following formula: V = πab^2^/6, where (a) is the longest diameter and (b) is the shortest diameter perpendicular to (a)^46^. Mice were euthanized at the end of the follow-up period or when they met criterion described at experimental endpoints. All experimental protocols were approved by Old Dominion University Institutional Biosafety Committee (IBC) and Institutional Animal Care and Use Committee (IACUC). And all experiments were performed in accordance with relevant guidelines and regulations.

### Electrode configuration

Two types of electrode configurations were used in the study: a 2-needle electrode with a 5 mm gap and a 4-needle electrode arranged in an array of 7 mm × 5 mm spacing. The electrodes were partially covered with polytetrafluoroethylene tubes to prevent surface flashover during *in vivo* treatment. The exposed area of the needles were the parts inserted and remained in the biological sample/tissue during treatment. For the purposes of the experiments in this study, the length of the insulating tubing was set to allow 3 mm of bare needle for insertion.

### Laser heating and control system

To achieve controlled heating and maintain a relatively constant surface temperature of the treatment object at 43 °C during the time of treatment, a programmable and automatically adjustable heating system was developed. The heating system consists of an infrared laser whose optical emission can be modulated in time, an optical fiber that delivers the infrared laser light to the treatment surface, a temperature sensor (a thermopile (ZTP-135SR) or a thermocouple) that reads the instantaneous surface temperature, and a programmable control circuit that provides the laser modulation as well as the temperature information to the readout such as a LabVIEW platform in a laptop or a computer. The surface temperature of the target is maintained by applying a time-modulated laser heating based on the temperature readout by the sensors. When the temperature measured by the sensor exceeds the desired temperature, the heating source shuts off and the heat loss due to heat exchange between the target tissue and the ambient environment will cause the temperature to go down. When the temperature of the target is below the set temperature, the laser irradiation is turned on again and the feedback loop repeats. This temporally modulated system utilizes short laser pulses and the rapid response time in millisecond time scale to achieve the desired temperature of the target tissue with precisions of ±2 °C.

### Electrical diagnostics

The square wave electroporation system listed in section “materials and instruments” provided pulses of 100 µs duration and up to 5 kV amplitude to the electrodes. Measurements of the voltage across the treated tissue area between the electrodes were performed by means of a voltage probe. The voltage probe consists of a resistive voltage divider with a divider ratio of 1:100. The current was measured by means of a current-viewing resistor, which was placed within the voltage probe and was calculated using Ohm’s Law. At the beginning of testing, the current limiting resistor was set to 50 Ohms but it was later discovered that this value gave low resolution results at lower input voltages (<1,500 kV). This resistor was then changed to 150 Ohm, which increased the resolution of the reading. Both, voltage and current signals were recorded with the digital oscilloscope. The ratio of the measured voltage and current provided information on the temporal change in impedance of the biological load. The pulsed voltage values, current and impedance were recorded in real time using a custom-designed LabVIEW program.

### *Ex vivo* IRE tumor ablation

A 3D agarose cell-culture model^47^ was adopted for *ex vivo* tumor ablation. Solutions of 1% and 2.5% low-gelling temperature agarose in complete media were prepared and kept at 37 °C. A base layer of 900 µL 2.5% low-gelling temperature agarose was applied to individual wells of a 12 well tissue culture plate. The plate was then chilled at 4 °C until addition of cell suspension. Briefly, 3 × 10^6^ Pan02 cells were resuspended in 1 mL of 1% low-gelling agarose and overlaid on the 2.5% layer. The plate was immediately returned to 4 °C for 4 minutes, after which the 3D cell models were incubated at 37 °C for 20 minutes.

Electric pulses were delivered to tumor using either a two-needle electrode or a four-needle electrode at either room temperature or 43 °C. A heat block was used to reach and maintain 43 °C. Temperature was monitored either with a thermocouple inserted in the edge of the agarose if cells were heated on the heat block, or with a thermopile integrated into the electrode adjacent to a fiber optic laser for those cells which were laser-heated. The pulse parameters were pulse duration 100 µs, frequency 1 Hz, 80 pulses, and applied electric fields of 750 V or 1500 V/cm for the two-needle electrode and 750 V/cm for the four-needle electrode.

### Quantification of *ex vivo* tumor ablation

Immediately after IRE treatment, 1 mL fresh media was added to each treated well, and the plate was transferred to an incubator at 37 °C with 5% CO_2_ for 1.5 hours. Propidium Idodide (PI, 4 µg/ml) was then added to the cell culture media and returned to the incubator. After 30 minutes of staining, culture media was replaced with DPBS and images of the ablation zone were obtained using a Leica MZFLIII fluorescence stereomicroscope (Leica) equipped with a Leica DFC420 C CCD camera. All images were taken at the same exposure parameters. Fluorescence images were quantified with ImageJ software (downloaded from imagej.nih.gov/ij/). Quantification of ablation zone was reported as integrated fluorescence density in relative fluorescence units (RFUs).

### Electric field modeling of the needle-electrodes

A two-dimensional electrostatic model was compiled using a Comsol Multiphysics AC/DC module. The electric field distribution of a four-needle electrode configuration immersed in water was calculated. Water was used in the simulation as the medium here since the agarose gel can be assumed to have high content of water and its dielectric property is close to water (relative permittivity; conductivity μS/m). An area of 20 × 20 mm^2^ that is more than one order of magnitude higher than the needle array was used for the computation to ensure the boundary effect (if any) has negligible impact on the field mapping results. The field distribution is obtained by solving the finite element AC/DC model under a steady state condition where the voltage across the anode (the left two needles) and cathode (the right two needles) is constant.

### *In vivo* IRE treatment

For *in vivo* IRE tumor ablation, EPs were delivered to tumor tissue using a four needle electrode array (Fig. 1) with 5 × 7 mm gaps when tumor size reached 8–10 mm in diameter with an average tumor volume of 250–300 mm^3^. There is a 7 mm gap between the anode and the cathode pins and a 5 mm gap between the two anodes or the two cathodes. The pulse parameters were pulse duration 100 µs, frequency 1 Hz, pulse number 90 and applied electric fields 750 V/cm to 2500 V/cm, dependent on experimental design. Carprofen (Rimadyl, 5 mg/kg) was given subcutaneously immediately before procedure and every 24 hours for 4 days to prevent/reduce pain caused by tumor necrosis.

### Statistical analysis

All values are presented as the mean ± standard deviation (SD). Analysis of tumor volume at different time points will be completed by One Way ANOVA. Animal survival was analyzed with Kaplan–Meier Survival Analysis (LogRank test). 2-tailed Student’s t-test (2 groups) was utilized to analyze quantitative *ex vivo* tumor ablation and *in vivo* tumor temperature increase due to IRE. Statistical significance is assumed at p < 0.05. All statistical analysis including Kaplan-Meier Survival Analysis will be completed using the SigmaPlot 12.0 (Aspire Software International).

## Electronic supplementary material

Supplementary Materials
