# Supplementary material for: Controllable Moderate Heating Enhances the Therapeutic Efficacy of Irreversible Electroporation for Pancreatic Cancer
Source: Sci Rep. 2017 Sep 18;7:11767. doi: 10.1038/s41598-017-12227-4 (PMC5603521; doi:10.1038/s41598-017-12227-4)
Supplement: Supplementary file 1 — Supplementary Materials [file 41598_2017_12227_MOESM1_ESM.doc]

**Title:**

**Controllable Moderate Heating Enhances the Therapeutic Efficacy of Irreversible Electroporation for Pancreatic Cancer**

Authors:

Chelsea M. Edelblute^1,+^, James Hornef^2,+^, Niculina I. Burcus^1^, Thomas Norman^2^, Stephen J Beebe^1^, Karl Schoenbach^1^, Richard Heller^1^, Chunqi Jiang^1, 2^ and Siqi Guo^1,*^

**Affiliations:**

^1^ Frank Reidy Research Center for Bioelectrics, Old Dominion University. Norfolk, Virginia 23508, USA.

^2^ Department of Electrical & Computer Engineering, Batten College of Engineering & Technology, Old Dominion University. Norfolk, Virginia 23508, USA.

^+^ These authors contributed equally to this work.

* Correspondence to: Siqi Guo. Email: [s2guo@odu.edu](mailto:s2guo@odu.edu)

**Supplementary Materials**

**Supplementary Figures and Figure Legends:**


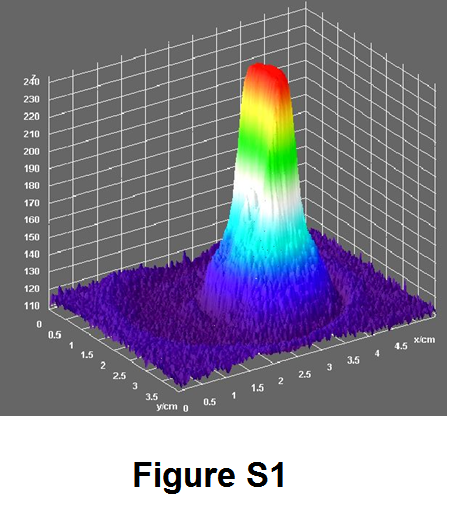


**Fig. S1. 3D heat profile of laser heating during pig skin calibration.**

This 3D laser profile was taken from the experimental results of the pig skin laser profile calibration. The sample was heated continuously for a total of 5 minutes and a thermal image was taken every 10 seconds, with the thermal camera about 25 cm from the sample. Image shown is at 160 seconds. ImageJ software was used to extrude the 2D image into 3D, with the intensity profile being from 22°C to 31°C.


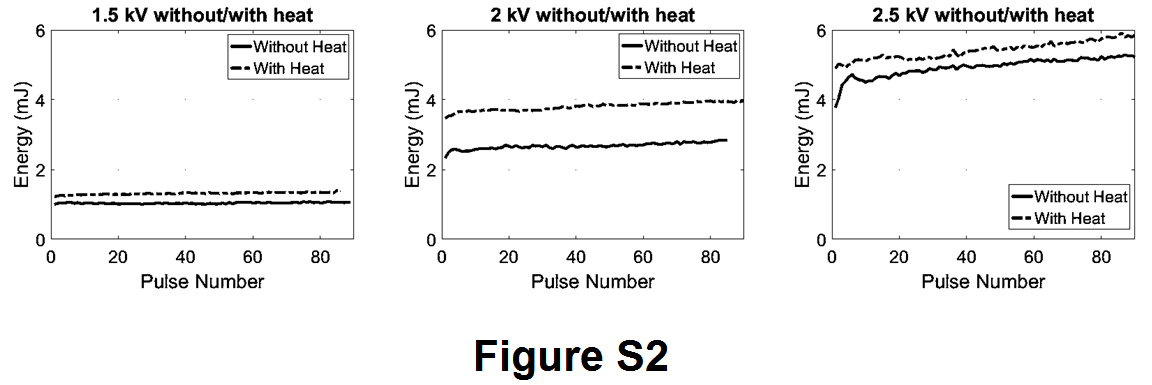


**Fig. S2. Energy per pulse measurement during MHIRE treatment**.

Using the voltage and current data collected from the probes, the energy per pulse trends were calculated. Each data point represents an average energy of 4 to 5 repeats for that pulse. IRE parameters: 100 µs pulse width, 90 pulses, frequency of 1 Hz and applied electric fields 1.5 kV/cm (left), 2 kV/cm (middle) and 2.5 kV/cm (right).
